# Measles and Rubella Elimination Trends in the WHO Eastern Mediterranean Region, 2023–2024: Progress and Recent Epidemiological Shifts

**DOI:** 10.3390/vaccines14080659

**Published:** 2026-07-27

**Authors:** Muhammad Farid, Mohammad Nadir Sahak, Isaac Baffoe-Nyarko, Amany Ghoniem, Sharifuzamman, Eman Elmahdy, Quamrul Hasan, Mohammed Osama Mere, Palwasha Anwari

**Affiliations:** Department of Polio Eradication and Vaccine Pervertible-Diseases, World Health Organization Regional Office for the Eastern Mediterranean, Cairo 11371, Egypt; sahakm@who.int (M.N.S.); banyark@yahoo.co.uk (I.B.-N.); ghoniema@who.int (A.G.); sharifuzzamanm@who.int (S.); elmahdye@who.int (E.E.); quamrulh@gmail.com (Q.H.); mereo@who.int (M.O.M.); palwashanwari@gmail.com (P.A.)

**Keywords:** measles, rubella, elimination, MCV1, MCV2, Eastern Mediterranean Region (EMR)

## Abstract

**Background**: Measles and rubella remain key indicators of immunization system performance and are central to the Immunization Agenda 2030 (IA2030). Disruptions caused by the COVID-19 pandemic have widened immunity gaps and accelerated resurgence of vaccine-preventable diseases across multiple regions. This study assesses progress toward measles and rubella elimination in the WHO Eastern Mediterranean Region (EMR) during 2023–2024. **Methods**: We conducted a retrospective descriptive analysis of measles and rubella surveillance data, WHO/UNICEF Estimates of National Immunization Coverage (WUENIC), and Joint Reporting Form (eJRF) data for all 22 EMR countries and territories. Key indicators included disease incidence, MCV1 and MCV2 coverage, surveillance performance metrics, and verified elimination status. **Results**: Regional measles incidence increased from 110.8 to 116.3 per million population, while rubella incidence more than doubled from 1.9 to 4.3 per million. Large outbreaks in Yemen, Iraq, and Afghanistan disproportionally drove regional trends. MCV1 coverage remained stable at approximately 80%, while MCV2 coverage increased marginally from 73% to 75%. Only nine of 22 countries achieved ≥95% coverage for both doses in 2024. Persistent zero-dose populations, high dropout rates, and ongoing immunity gaps sustained transmission across multiple settings. **Conclusions**: The EMR remains off track to achieve measles and rubella elimination by 2030. Accelerated, equity-focused efforts are urgently needed to strengthen routine immunization systems, reduce zero-dose population, enhance surveillance capacity, and improve outbreak response—particularly in fragile and conflicted-affected settings.

## 1. Introduction

Measles and rubella (MR) remain leading causes of vaccine-preventable morbidity and mortality globally despite the availability of safe, effective, and affordable vaccines [1,2]. The Immunization Agenda 2030 (IA2030), endorsed during the World Health Assembly in 2020, sets global vision and strategy aiming to achieve Universal Health Coverage (UHC) and tackling zero-dose children [3]. Building on lessons learned from Global Vaccine Action Plan [4], IA2030 identifies measles incidence and vaccination coverage as a key indicator and tracer to improve immunization services and strengthen primary health care [3]. Measles serves as a “tracer” of health systems’ capacity to deliver essential childhood vaccines, underscoring the importance of: (a) robust measles surveillance systems to identify and monitor immunity gaps; (b) sustained high routine immunization coverage at least 95% coverage with 2 doses of measles-containing vaccine (MCV); and (c) the ability of health systems to effectively reach zero-dose and under-immunized children. The Global Measles-Rubella Strategic Framework 2021–2030 [5], and the Global Measles Outbreaks Strategic Response Plan 2021–2023 [6] align with IA2030 to accelerate measles and rubella elimination. At its 72nd session (RC 72), the WHO Regional Committee for the Eastern Mediterranean Region (EMR) endorsed regional rubella elimination goals [7]. Accordingly, all 22 EMR countries in WHO EMR are committed to achieving measles and rubella elimination through sustained high population immunity, sensitive case-based surveillance, and timely outbreak detection and response. Measles and rubella (MR) elimination is defined as the absence of endemic transmission for at least 12 months, verified by a high-quality surveillance system, and constitutes a critical marker of health system performance and equity [8].

The WHO EMR comprises 22 countries and territories with substantial heterogeneity in socioeconomic conditions, health system capacity, and political stability. Over the past decade, the Region has made important progress toward measles and rubella elimination, with several countries achieving or sustaining elimination status and strengthening laboratory-supported surveillance networks [9,10]. However, persistent challenges including protracted conflicts, population displacement, recurrent humanitarian emergencies, weak routine immunization performance in some settings, and the impact of the COVID-19 pandemic have resulted in widening immunity gaps and recent outbreaks [11,12].

Against this backdrop, the 2023–2024 period marked a critical juncture for measles and rubella (MR) elimination in EMR, characterized by post-pandemic resurgence [11,13], widening immunity gaps [11], recurrent outbreaks, and persistent health system fragility [14,15,16]. This study provides a comprehensive regional assessment of epidemiological trends, immunization coverage, surveillance performance, and outbreak dynamics to evaluate progress toward MR elimination targets and identify critical priorities for accelerating elimination efforts across the region.

## 2. Methods

### 2.1. Data Sources

We conducted a retrospective descriptive analysis of measles and rubella epidemiology across all 22 countries and territories in the WHO EMR from January 2023 to December 2024. Data were obtained from three primary sources: (1) the WHO EMR measles and rubella surveillance dashboard, which aggregates case-based surveillance data from monthly country reports; (2) the WHO/UNICEF Estimates of National Immunization Coverage (WUENIC) for standardized national coverage estimates, and; (3) the electronic Joint Reporting Form (eJRF), which contains country-reported annual immunization and surveillance data.

Countries were stratified by (1) measles incidence levels and (2) immunization performance (MCV1 and MCV2 coverage), to better interpret heterogeneity in transmission and programme performance.

These sources provided data on reported measles and rubella cases, routine immunization coverage, and supplemental immunization activities (SIA) across the region. All 22 EMR countries and territories were included in the analysis.

### 2.2. Case Definitions and Key Indicators

All countries and territories have applied standard WHO case definitions for measles and rubella. A suspected case was defined as any individual with fever and maculopapular rashes, or when a health care provider suspects it as measles or rubella. Confirmed cases were classified as laboratory-confirmed based on IgM positivity, epidemiologically linked, or clinically compatible in the absence of adequate laboratory investigation. Confirmed cases were further categorized as endemic, imported, or import related [17].

Key indicators were selected in accordance with the WHO measles-rubella elimination monitoring framework and were used to assess disease burden, population immunity, immunization programme performance, and surveillance quality across the Region. These indicators included:

- Measles and rubella incidence: Annual incidence of confirmed cases per 1,000,000 population, used to assess disease burden and progress toward elimination targets.
- Surveillance sensitivity: Measured by the annualized non-measles, non-rubella discarded case rate per 100,000 population, reflecting the sensitivity and performance of the surveillance system in detecting suspected cases.
- Immunization coverage: National coverage estimates for the first and second doses of measles containing vaccine (MCV1 and MCV2), based on the 2024 WUENIC [18].
- Supplementary immunization activities (SIAs): Information on the number, scope, target populations, and reported administrative coverage of SIAs implemented during 2023–2024.

Achieving and sustaining high population immunity, defined as at least 95% coverage with both MCV doses through routine immunization services, complemented by high-quality SIAs, is considered essential for interrupting endemic measles and rubella transmission and sustaining elimination.

### 2.3. Analysis Approach

A descriptive analytical approach was employed to examine temporal trends, geographic variation, and programme performance related to measles and rubella elimination across the WHO Eastern Mediterranean Region (EMR) during 2023–2024. Analyses focused on changes in disease incidence, immunization coverage, surveillance sensitivity, outbreak occurrence, and supplementary immunization activities (SIAs) at both regional and country levels. Where data were available, monthly trends were also assessed to better characterize outbreak dynamics and temporal fluctuations in transmission.

To better contextualize regional heterogeneity and differences in programme performance, countries were stratified using two analytical frameworks: (1) measles incidence levels (categorized as <1, 1–4.9, 5–19.9, 20–99.9, and ≥100 cases per million population) and (2) immunization performance, based on MCV1 and MCV2 coverage relative to the ≥95% elimination threshold.

The analysis also incorporated comparative assessment of regional and country-level trends across key indicators, including incidence rates, surveillance sensitivity, vaccination coverage, and SIA implementation, to identify patterns associated with progress toward or setbacks in elimination goals.

All quantitative analyses and data visualizations were conducted using R software (R Foundation for Statistical Computing, Vienna, Austria, Version 4.5.1.) and Microsoft Excel (Microsoft Corporation, Redmond, WA, USA). Findings were synthesized and presented through tables, figures, and regional summaries to illustrate trends, disparities, and emerging priorities for measles and rubella elimination in the EMR.

## 3. Results

### 3.1. Regional Trends in Measles and Rubella Incidence

The WHO EMR experienced a continued resurgence of measles transmission during 2023–2024. Regional measles incidence increased from 110.8 to 116.3 per 1,000,000 population, reflecting sustained transmission across several high-burden settings (Table 1). In parallel, rubella incidence more than doubled during the same period, rising from 1.9 to 4.3 per 1,000,000 populations, indicating emerging gaps in rubella immunity (Table 1).

Surveillance sensitivity improved across the region. The annualized non-measles and non-rubella discarded case rate increased from 6.0 to 7.2 per 100,000 populations exceeding the WHO recommended benchmark in both years (Table 1, Panel C).

### 3.2. Country-Level Heterogeneity and Outbreak Patterns

**Figure 1:**
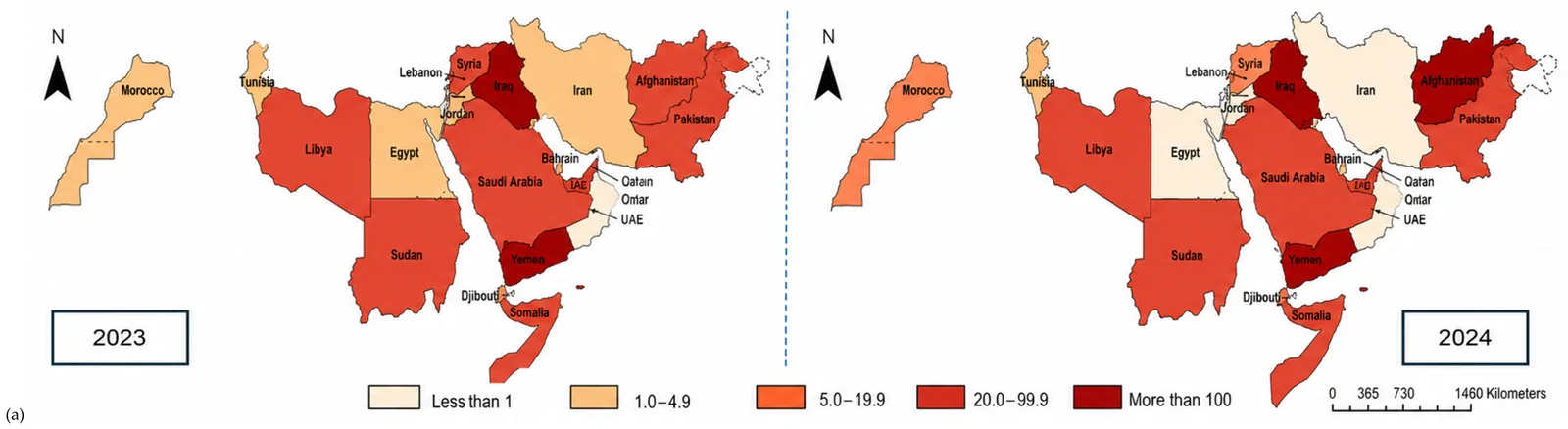

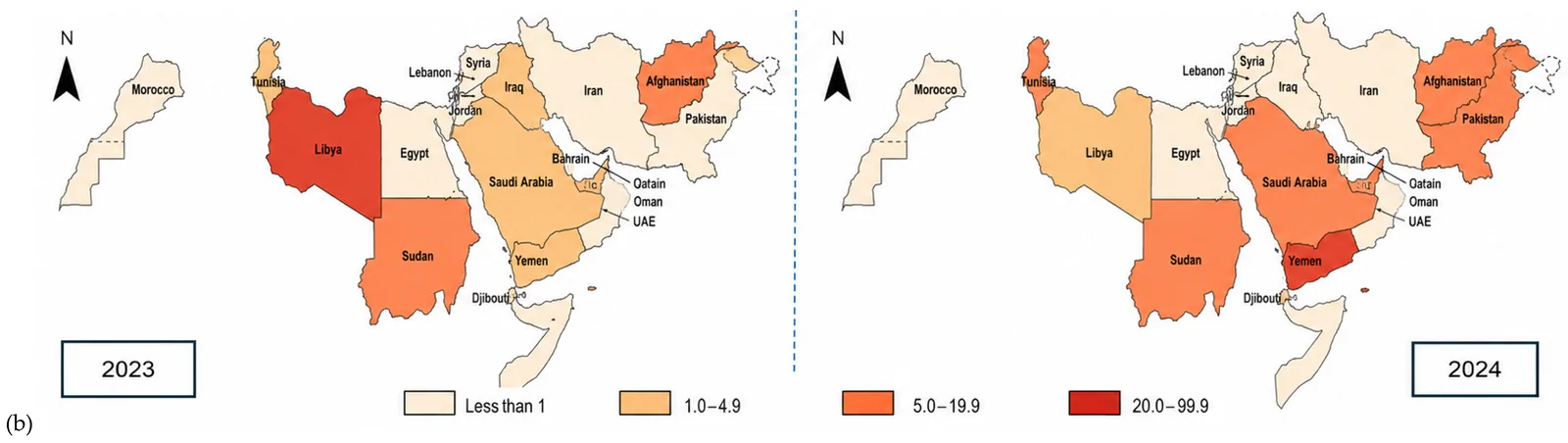
(**a**) Measles incidence rates per 1,000,000 population in WHO Eastern Mediterranean Region (EMR) countries, 2023 and 2024. Countries are categorized by incidence thresholds (<1, 1.0–4.9, 5.0–19.9, 20.0–99.9, ≥100) Maps illustrate geographic distribution of measles burden. *Source: WHO EM Regional surveillance data shared by member states*. (**b**) Rubella incidence rates per 1,000,000 population in WHO Eastern Mediterranean Region (EMR) countries, 2023 and 2024. Countries are categorized by incidence thresholds (<1, 1.0–4.9, 5.0–19.9, 20.0–99.9, ≥100). Maps illustrate geographic distribution of measles burden. *Source: WHO EM Regional surveillance data shared by member states*.

Substantial heterogeneity in measles epidemiology was observed across countries, with a small number of high-burden countries disproportionately driving regional transmission trends (Table 1, Panel A; Figure 1a). In 2023, the highest measles incidence rates per 1,000,000 population were reported in Yemen (1258.4), followed by Iraq (214.0), Somalia (97.0), Sudan (81.9) and Pakistan (70.8).

By 2024, the epidemiological profile shifted considerably. Iraq experienced the most pronounced resurgence, with measles incidence increasing more than threefold to 703.1 per 1,000,000 population, representing the largest relative increase among high-burden countries (Table 1, Panel A). Afghanistan also recorded a substantial rise, reaching 229.1 per 1,000,000 population, highlighting persistent transmission and vulnerability in fragile and conflict-affected settings. Although incidence declined in Yemen and Somalia during 2024, both countries continued to contribute substantially to the regional measles burden, with Yemen remaining the highest-burden setting in the Region despite a 53% reduction compared with 2023. Pakistan maintained sustained high transmission across both years, with incidence increasing from 70.8 to 96.6 per 1,000,000 population.

Overall, the regional epidemiological profile during 2023–2024 was shaped primarily by three major transmission settings: the prolonged outbreaks in Yemen and Pakistan, and the sharp resurgence observed in Iraq. Several countries that had previously reported relatively lower incidence, including Morocco and Qatar, also experienced increases in measles incidence during 2024, suggesting emerging immunity gaps and localized transmission risks. In contrast, substantial declines were observed in Lebanon, Syria, Sudan, and Jordan (Table 1, Panel A).

Rubella epidemiology similarly demonstrated considerable inter-country variation (Table 1, Panel B; Figure 1b). The regional increase in rubella incidence was driven primarily by a sharp surge in Yemen, where incidence rose from 4.7 to 65.7 per 1,000,000 population between 2023 and 2024, representing an approximately fourteen-fold increase. Libya reported the highest rubella incidence in 2023 (17.7 per 1,000,000 population), and the second highest in 2024 (5.3 per 1,000,000 population), after Yemen. Afghanistan, Djibouti, and Saudi Arabia also reported relatively elevated rubella incidence during 2024, suggesting ongoing susceptibility and immunity gaps in several settings.

Surveillance performance also varied considerably across countries (Table 1, Panel C). While the regional discarded case rate exceeded the WHO-recommended benchmark in both years, several countries—including Sudan, Morocco, Oman, Kuwait, and Tunisia—did not achieve the minimum target of ≥2 discarded non-measles, non-rubella cases per 100,000 population in 2024, indicating potential surveillance sensitivity gaps. In contrast, Bahrain, Pakistan, Palestine, Afghanistan, and the United Arab Emirates reported particularly high discarded case rates, reflecting comparatively strong surveillance sensitivity and case investigation capacity.

### 3.3. Temporal Trends in Transmission

**Figure 2:**
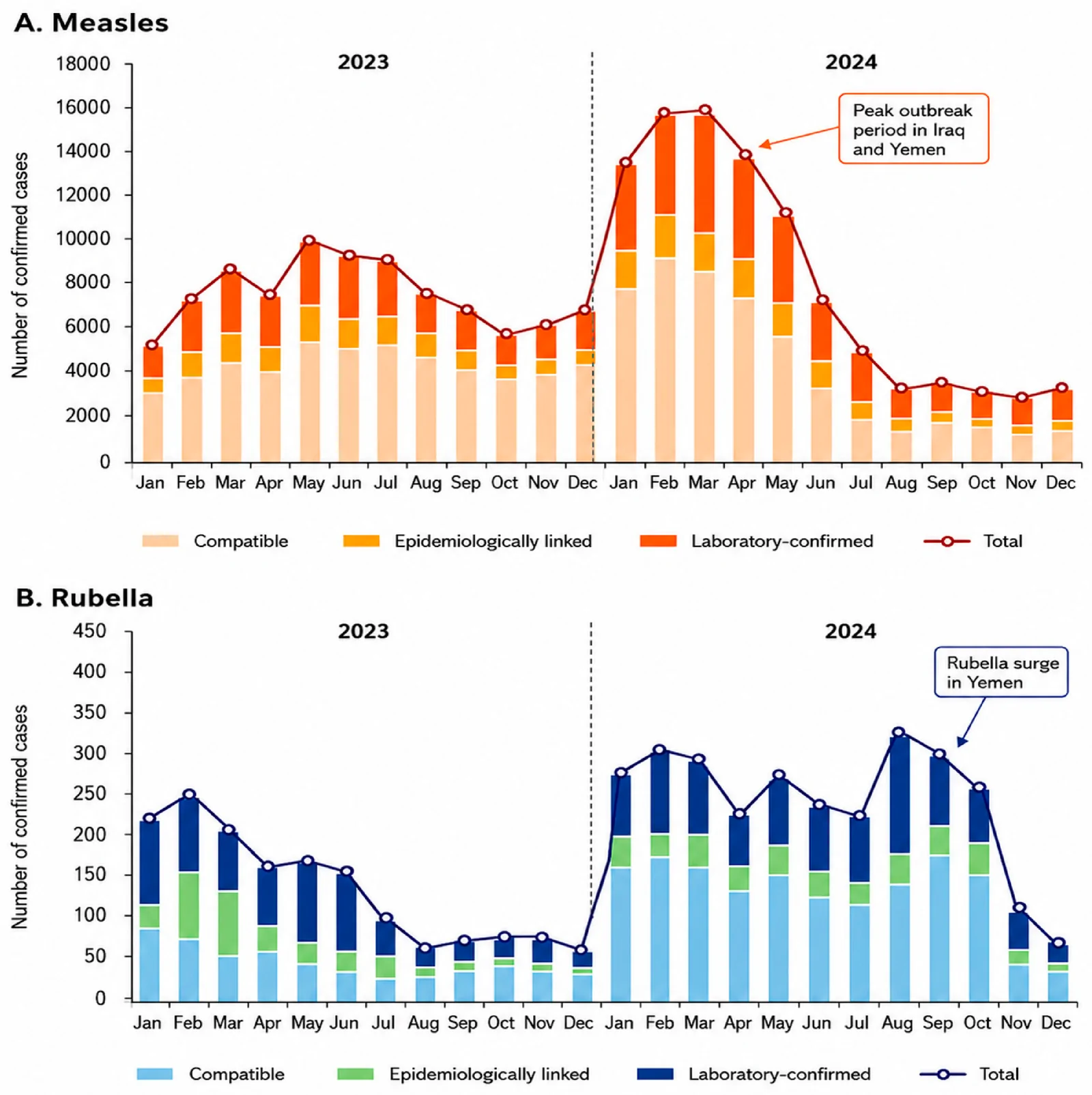
Monthly distribution of confirmed measles and rubella cases in the WHO Eastern Mediterranean Region (EMR), 2023–2024, by case classification. (**A**) Monthly trend of confirmed measles cases and (**B**) monthly trend of confirmed rubella cases reported in the EMR during 2023–2024. Stacked bars represent cases classified as laboratory-confirmed, epidemiologically linked, and clinically compatible, while overlaid line graphs indicate the total number of confirmed cases by month. The figures illustrate marked temporal variation in transmission patterns, including major measles outbreaks in Iraq and Yemen and the substantial rubella surge observed in Yemen during 2024.

Monthly trends demonstrated distinct epidemic and seasonal transmission patterns from both measles and rubella across the WHO EMR during 2023–2024 (Figure 2).

In 2023, measles transmission remained relatively sustained throughout the year, with moderate monthly fluctuations and increased activity during half of the year. Reported monthly measles cases generally ranged between approximately 6000 and 10,000 cases, with peaks observed during the mid-year months (Figure 2A).

In contrast, 2024 was characterized by a marked regional resurgence during the first quarter of the year. Monthly measles cases increased sharply beginning in January and peaked in February–March 2024, reaching approximately 15,000–16,000 cases per month. This surge coincided with major outbreaks in Iraq and Yemen and contributed substantially to the elevated regional incidence observed in 2024. Following the early-year peak, measles transmission declined progressively during the second half of the year, from August through December 2024, monthly case counts fell below all levels recorded in 2023, representing the lowest monthly totals of the entire 2023–2024 period (Figure 2A).

The composition of reported measles cases also varied over time. Clinically compatible cases constituted the majority of reported cases throughout the period, while laboratory-confirmed cases contributed a smaller but consistent proportion, particularly during outbreak periods (Figure 2A). This pattern reflects continued reliance on clinical case ascertainment and highlights the need to strengthen laboratory-based confirmation capacity, particularly in high-burden countries where case volumes during outbreak peaks outstripped testing capacity.

Rubella transmission remained substantially lower than measles transmission throughout the study period, with monthly reported cases generally remaining below 400 cases per month (Figure 2B). Nevertheless, rubella displayed a noticeable upward trend during 2024, consistent with the increase in annual regional incidence. Elevated monthly rubella activity was particularly evident during mid- to late-2024 and was largely driven by the sharp increase in cases reported from Yemen. Similar to measles, clinically compatible cases represented the majority of reported rubella cases, with laboratory-confirmed cases accounting for a smaller share during periods of increased transmission (Figure 2B).

### 3.4. Age Distribution and Vaccination Status of Reported Measles Cases

**Figure 3:**
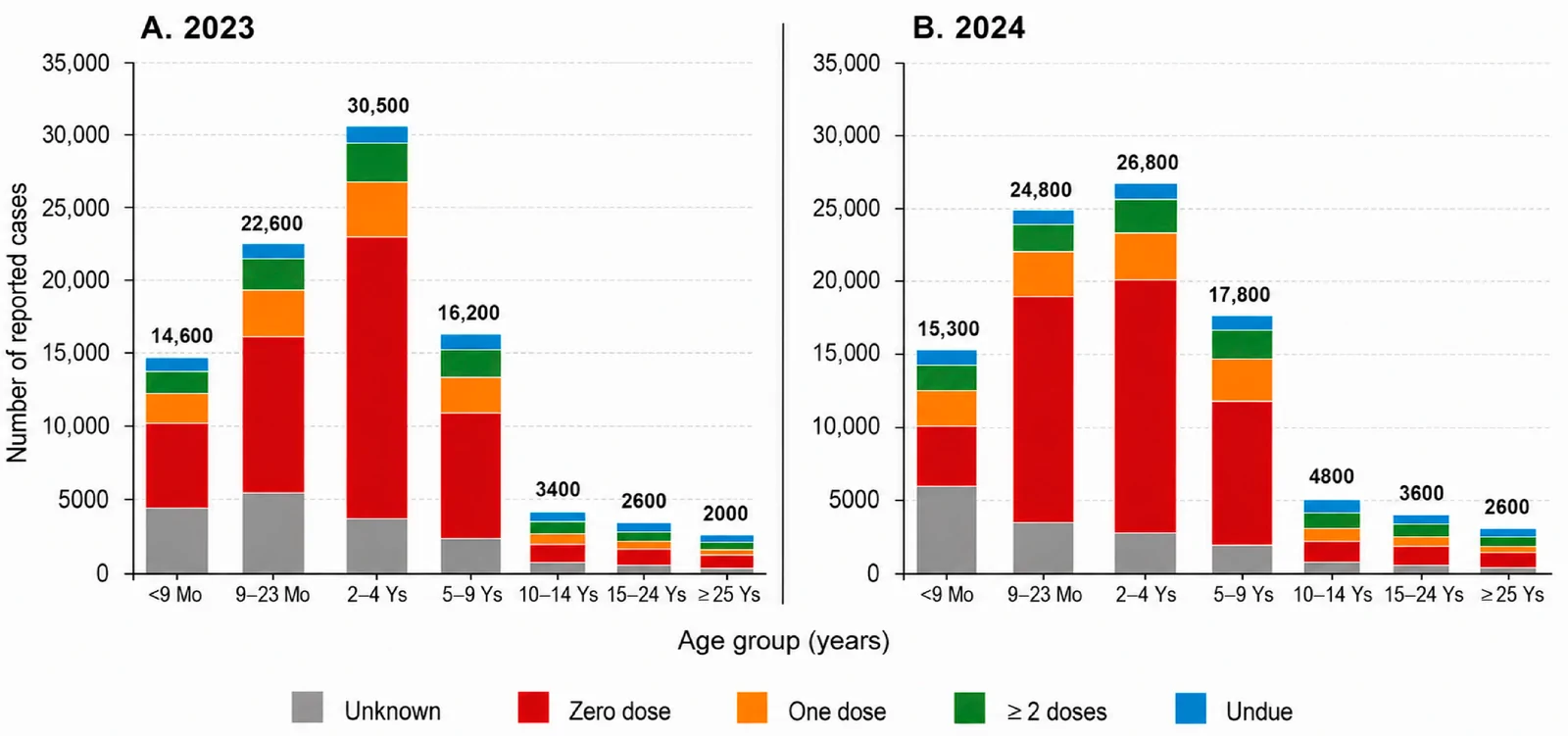
Distribution of reported measles cases by vaccination status across age groups in the WHO Eastern Mediterranean Region (EMR), 2023–2024. (**A**) Reported measles cases in 2023. (**B**) Reported measles cases in 2024. Bars show distribution of cases by age group and vaccination status (unknown, zero dose, one dose, ≥2 doses, and undue). (Source: Regional measles and rubella surveillance database.)

The age distribution of reported measles cases revealed persistent immunity gaps across multiple age groups in the WHO EMR during 2023–2024 (Figure 3). Among cases with reported age and vaccination status (*n* = 111,760), the majority occurred among children under five years of age (72.9%, *n* = 81,473). Children aged 9–23 months and 2–4 years accounted for the largest proportion or reported cases in both years (Figure 3). Notably, a substantial proportion of cases within these age groups were reported among zero-dose children and those who had received only one vaccine dose, despite being eligible for routine measles-containing vaccination.

Infant aged <9 months also represented a considerable portion of reported cases. As this age group is below the recommended age for the first dose MCV, these cases likely reflect intense community transmission and inadequate indirect protection resulting from insufficient population immunity. Older age groups also contributed substantially to transmission. Approximately 11% (*n* = 12,294) of reported cases occurred among individuals aged ≥10 years, suggesting accumulated susceptibility over time.

Zero-dose cases were particularly concentrated among children aged 9–23 months and 2–4 years, while one-dose cases accounted for a large proportion of reported infections across nearly all age categories, including incomplete vaccination and insufficient population immunity. In contrast, cases among individuals reporting receipt of ≥2 vaccine doses remained comparatively lower across all age groups.

At the same time, a notable proportion of zero-dose cases persisted among older children and adolescents, indicating long-standing gaps in immunization coverage. Among individuals aged ≥15 years, a high proportion of cases had unknown vaccination status, reflecting limitations in routine vaccination-status ascertainment among adults.

### 3.5. Immunization Coverage and Immunity Gaps

Routine immunization coverage remained below the threshold required for measles elimination in many countries across the WHO EMR during 2023–2024. Regional coverage for MCV1 coverage remained relatively stable at approximately 80% in both years, while MCV2 coverage increased slightly from 73% to 75% (Table 2).

In 2024, only nine of 22 countries achieved ≥95% coverage for both MCV1 and MCV2 (Table 2). More than half of countries remained below this threshold for at least one dose, particularly for MCV2 coverage. Countries with the lowest routine coverage included Yemen, Sudan, Afghanistan, Somalia, and Lebanon, reflecting the continued impact of fragility, conflict, displacement, health system disruption, and limited service access on immunization programme.

Marked disparities between MCV1 and MCV2 coverage were also observed across several countries, highlighting weaknesses in programme continuity and completion of the two-dose vaccination schedule. The largest MCV1–MCV2 dropout rates in 2024 were observed in Somalia (50.0%), Djibouti (36.0%), Sudan (21.7%), and Afghanistan (20.0%) (Table 2 Panel B). Although some countries demonstrated improvements in coverage between 2023 and 2024, notably Libya, Jordan, and the Syrian Arab Republic, coverage remained suboptimal in many settings and insufficient to close existing immunity gaps.

### 3.6. Immunization Programme Performance and Population Immunity

During 2023 and 2024, countries across the WHO EMR implemented large-scale supplementary immunization activities (SIAs) and outbreak response vaccination campaigns in response to recurrent measles outbreaks and persistent immunity gaps. Approximately18 million children received additional dose of measles-and rubella-containing vaccines (MRCV/MCV) through SIAs and reactive vaccination campaigns conducted in Djibouti, Iraq, Pakistan, and Sudan.

### 3.7. Surveillance and Laboratory Performance

#### 3.7.1. Case-Based Surveillance Performance and Sensitivity

Surveillance performance improved in several areas during 2023–2024, although heterogeneity persisted across countries (Table 3). The proportion of countries achieving timely and complete investigation of suspected measles cases increased from 41% in 2023 to 50% in 2024, suggesting gradual strengthening of case investigation capacity across the Region. Similarly, most countries maintained strong laboratory specimen collection and testing performance, with over 90% of countries achieving the target for adequate specimen collection and testing in proficient laboratories during both years.

However, performance declined for several key surveillance sensitivity indicators. The proportion of countries reporting ≥80% of IgM laboratory results to national authorities within four days declined from 64% in 2023 to 50% in 2024, indicating challenges in timely laboratory reporting and information flow. In addition, the proportion of countries achieving the recommended annualized discarded non-measles/non-rubella case rate ≥ 2 per 100,000 population declined slightly from 73% to 68%, reflecting persistent surveillance sensitivity gaps in several settings.

Although regional surveillance sensitivity indicators remained above WHO minimum benchmarks overall, under-detection of cases and delayed reporting continued to affect several countries, particularly those experiencing insecurity, population displacement, and weak health system capacity. These surveillance limitations likely contributed to delayed outbreak detection and underestimation of disease burden in some fragile and conflict-affected settings.

#### 3.7.2. Laboratory Confirmation and Virologic Surveillance

All 22 countries and territories in the Region utilized enzyme-linked immunosorbent assay (ELISA) as the standard method for MR laboratory confirmation. Overall laboratory performance remained strong, with nearly all laboratories achieving satisfactory scores in recent serology proficiency testing exercises.

Progress in molecular surveillance and genotyping capacity was evident but remained uneven across the Region. Eleven countries regularly submitted sequence data to the WHO Measles Nucleotide Surveillance (MeaNS) database. Nine countries—Bahrain, Egypt, Jordan, Oman, Pakistan, Qatar, Saudi Arabia, Syria, and Tunisia—have functional in-country genotyping and sequencing capacity and consistently generate high-quality sequence data. Afghanistan and Libya continued to rely on Regional Reference Laboratories (RRLs) to compensate for limited national sequencing capacity.

Important gaps in virologic surveillance persisted. Eight countries did not regularly submit sequencing data to MeaNS, limiting regional ability to comprehensively track transmission pathways and identify importation patterns. Somalia, Djibouti, Sudan, and Yemen required substantial capacity strengthening in molecular detection, sequencing, genotyping, and data reporting. Iraq, Kuwait, Jordan, and the Syrian Arab Republic had trained personnel but lacked essential sequencing equipment, although installation of Sanger sequencing platforms was anticipated in Jordan and Syria. Lebanon, Morocco, and Palestine demonstrated improving capacity but still required refresher training and continued technical support to sustain sequencing quality and reporting consistency.

Sequencing capacity expanded during the study period. Molecular surveillance activities remained concentrated primarily during large outbreaks, resulting in underrepresentation of sporadic transmission chains and incomplete genotype characterization. Genotype B3 remained the most frequently detected measles strain across the Region, suggesting ongoing endemic transmission in multiple countries. Genotype D8 was detected less frequently and was more commonly associated with imported or import-related transmission chains.

### 3.8. Verification of Elimination and Progress Toward Targets

At the December 2025 meeting of the Regional Verification Commission (RVC) for measles and rubella, four countries, Bahrain, Egypt, Iran, and Oman, were verified as having sustained measles and rubella elimination status for 2024. Kuwait and Qatar were classified as having achieved measles and rubella elimination for 2024.

## 4. Discussion

The findings indicate that the WHO Eastern Mediterranean Region (EMR) is experiencing a setback in progress toward measles and rubella elimination, with increasing incidence and widening immunity gaps during 2023–2024. This resurgence is consistent with recent regional and global analyses showing rising measles incidence following COVID-19–related disruptions to immunization services and surveillance systems [11,12]. Although surveillance sensitivity has improved—as reflected by higher non-measles discarded case rates—this has not translated into interruption of transmission, underscoring the need for concurrent strengthening of both immunization and surveillance functions [19].

The resurgence is driven primarily by large outbreaks in high-burden and fragile settings, including Yemen, Iraq, and Afghanistan, where health system disruptions, conflict, and population displacement undermine routine immunization and outbreak response capacity. These findings reinforce the established role of measles as a tracer of health system performance under IA2030, reflecting inequities in access and delivery of essential health services [14]. Global evidence consistently demonstrates that measles incidence is disproportionately higher in low-income and crisis-affected countries, underscoring the link between system fragility and outbreak risk [13,14,19].

Routine immunization coverage in the region remains insufficient to sustain elimination. WHO recommends achieving ≥95% coverage with two doses of measles-containing vaccine (MCV1 and MCV2) to interrupt transmission; however, the majority of EMR countries remain below this threshold [5,20]. Persistent gaps between MCV1 and MCV2 coverage, as reflected in high dropout rates, indicate weaknesses in programme continuity and the delivery of second-year-of-life services [21,22]. These coverage deficits, combined with the persistence of large zero-dose population, continue to fuel susceptibility and endemic sustain transmission.

The age distribution of cases further highlights underlying immunity gaps. The concentration of cases among children under five years—including infants too young to be vaccinated—suggests both persistent missed vaccination opportunities and a high force of infection. Similar patterns have been observed in other settings, where intense transmission exposes unvaccinated infants and reflects suboptimal herd immunity [19,23,24]. The substantial burden among older children and adolescents further indicates the accumulation of susceptible cohorts over successive years of below-threshold coverage.

The observed increase in rubella incidence, particularly in outbreak-affected countries such as Yemen, raises concern for congenital rubella syndrome (CRS). Rubella infection during early pregnancy is associated with miscarriage, fetal death, or severe congenital anomalies, emphasizing the critical importance of maintaining high population immunity through vaccination [25,26,27]. These findings underscore the need to strengthen integrated measles–rubella control strategies to prevent CRS and accelerate progress toward elimination, particularly in settings with high incidence and limited access to antenatal care.

Longer-term regional trends show some encouraging improvements in immunization programme performance, including modest reductions in MCV1–MCV2 dropout and a decline in the number of children missing the second dose. Nonetheless, the persistence of large zero-dose populations indicates that access barriers, equity gaps, and system-level weaknesses remain unresolved. These findings align with global trends showing stagnating coverage and increasing population susceptibility despite incremental gains in some indicators [9,10,21], reinforcing the need for a more fundamental transformation of immunization programme delivery rather than incremental adjustments.

Laboratory confirmation and virologic surveillance remain essential components of elimination programmes, enabling confirmation of transmission chains and identification of importations. WHO guidelines emphasize the importance of high-quality surveillance systems integrating epidemiological and laboratory evidence for documenting elimination status [8,28]. However, operational gaps in specimen collection, transport, sequencing capacity, and timely reporting persist in several countries, limiting comprehensive characterization of transmission and delaying outbreak detection.

Persistent structural and operational challenges continue to impede progress toward measles and rubella elimination in the EMR. Conflict, insecurity, population displacement, and humanitarian emergencies disrupt routine immunization and delay outbreak response, particularly in hard-to-reach and underserved populations [14,15,16]. At the same time, health system constraints, including limited workforce capacity, insufficient domestic financing, and gaps in service delivery, affect programme quality, equity, and sustainability [12,29]. Challenges related to data quality, completeness, and timeliness hinder accurate assessment of population immunity and programme performance, potentially obscuring the true extent of immunity deficits [30].

Overall, these findings suggest that progress toward elimination remains uneven and fragile, requiring intensified and targeted efforts. Strategies should prioritize reaching zero-dose and under-immunized populations through equity-focused approaches, strengthening routine immunization systems, and enhancing surveillance and outbreak response capacity—especially in fragile and high-risk settings. The 2030 elimination targets are achievable only if the Region transitions from reactive outbreak response to proactive, sustained population immunity building.

This analysis has several limitations. First, data quality and completeness vary across countries, particularly in fragile and conflict-affected settings, where reporting may be inconsistent. Second, surveillance data almost certainly underestimate true incidence due to under-reporting, health-seeking limitations, and access constraints. Third, discrepancies between administrative coverage data and WUENIC estimates may affect interpretation of immunization performance. Despite these limitations, this analysis provides a comprehensive regional overview of trends using standardized WHO data sources and validated methodologies.

### Policy and Strategic Considerations

Efforts toward measles and rubella elimination in the EMR should remain aligned with the Immunization Agenda 2030 (IA2030) and global measles–rubella strategic framework. Strengthening cross-border collaboration, data sharing, synchronized vaccination campaigns, and coordinated outbreak response is essential to reduce the risk of virus importation across porous borders and mobile populations. Regional partners should accelerate support for zero-dose reduction strategies, increase domestic investment in immunization, and integrate elimination priorities into broader primary health care strengthening agendas.

Countries should prioritize high-burden, fragile, and conflict-affected settings through tailored approaches that integrate immunization with humanitarian response frameworks. Key priorities include restoring and sustaining routine immunization services, strengthening surveillance and laboratory systems, reducing zero-dose populations through community engagement and targeted outreach, and improving equitable access to vaccination in underserved and hard-to-reach communities. Progress toward elimination will require sustained political commitment, adequate resourcing, and multisectoral collaboration across health, humanitarian, and development actors.

## 5. Conclusions

The WHO Eastern Mediterranean Region remains off track to achieve measles and rubella elimination by 2030. Despite progress in some countries and improvements in surveillance sensitivity, ongoing outbreaks driven by large-scale transmission in fragile settings, persistently low immunization coverage, and substantial zero-dose populations continue to threaten regional gains and impede progress toward elimination targets.

Accelerated and targeted action is urgently required. Priorities include strengthen routine immunization systems, reducing zero-dose populations through equity-focused outreach, enhance surveillance and laboratory capacity, and ensuring timely and effective outbreak response. Success will depend on sustained political commitment, adequate financing, and coordinated action by national governments, regional partners, and international donors—underpinned by the recognition that measles and rubella elimination is both an achievable public health goal and a fundamental marker of health system equity and resilience.

## Figures and Tables

**Table 1:** Measles and Rubella Epidemiological and Surveillance Indicators by Country, WHO Eastern Mediterranean Region (EMR), 2023–2024.

**Panel A. Measles Incidence per 1,000,000 Population**			
**Country**	**2023**	**2024**	**% Change**
EMR	110.8	116.3	+5
Afghanistan	60.9	229.1	+276.2
Bahrain	0	0	—
Djibouti	63.3	51.3	−19.0
Egypt	2.2	0	−100.0
Iran, Islamic Republic	1.4	0.2	−85.7
Iraq	214	703.1	+228.6
Jordan	11.3	0.5	−95.6
Kuwait	1	1.2	+20
Lebanon	59.9	12.7	−78.8
Libya	24.2	24.9	+2.9
Morocco	3.5	10	+185.7
Oman	0	0	—
Pakistan	70.8	96.6	+36.4
Palestine	0.4	0	−100.0
Qatar	5.4	9.8	+81.5
Saudi Arabia	64.9	35.9	−44.7
Somalia	97	79.8	−17.7
Sudan	81.9	28.5	−65.2
Syria	31.5	5.4	−82.9
Tunisia	1.6	1.3	−18.8
United Arab Emirates	36.7	39.4	+7.4
Yemen	1258.4	591.1	−53.0
**Panel B. Rubella Incidence per 1,000,000 Population**			
**Country**	**2023**	**2024**	**% Change**
EMR	1.9	4.3	+126.3
Afghanistan	5.5	3.6	−34.5
Bahrain	0	0	—
Djibouti	3.5	2.6	−25.7
Egypt	0	0.3	—
Iran	0.1	0.2	+100
Iraq	1	0.1	−90.0
Jordan	0.4	0.4	0
Kuwait	0	0.4	—
Lebanon	1.2	0.9	−25.0
Libya	17.7	5.3	−70.1
Morocco	1	0.7	−30.0
Oman	0	0	—
Pakistan	1	1.3	+30
Palestine	0	0	—
Qatar	0.3	0.3	0
Saudi Arabia	3.4	2.2	−35.3
Somalia	0.9	0	−100.0
Sudan	9.8	1.4	−85.7
Syria	0.8	1	25
Tunisia	2.3	2.9	26.1
United Arab Emirates	1.3	1.4	7.7
Yemen	4.7	65.7	1298
**Panel C. Surveillance Sensitivity: Non-Measles, Non-Rubella Discarded Case Rate per 100,000 Population**			
**Country**	**2023**	**2024**	**WHO Target ≥ 2 Achieved (2024)**
EMR	6	7.2	Yes
Afghanistan	5.8	11.7	Yes
Bahrain	76.1	34.2	Yes
Djibouti	0.4	3.3	Yes
Egypt	2.8	2.5	Yes
Iran	8.1	8.2	Yes
Iraq	5.4	3.4	Yes
Jordan	6.5	3.9	Yes
Kuwait	2.7	1.6	No
Lebanon	6.7	2.3	Yes
Libya	21.8	8.1	Yes
Morocco	1	1.5	No
Oman	1.7	1.3	No
Pakistan	25.7	22.4	Yes
Palestine	7.8	12.7	Yes
Qatar	1.4	3.3	Yes
Saudi Arabia	9.3	6.3	Yes
Somalia	6.5	8.5	Yes
Sudan	0.6	0.3	No
Syria	5.3	3.7	Yes
Tunisia	1.5	1.3	No
United Arab Emirates	6.6	10	Yes
Yemen	3.8	2.1	Yes

**Table 2:** Coverage with the First and Second Doses of Measles-Containing Vaccine (MCV1 and MCV2) by Country, WHO Eastern Mediterranean Region (EMR), 2023–2024.

**Panel A. Immunization Coverage and Dropout, 2023**				
**Country**	**MCV1 (%)**	**MCV2 (%)**	**MCV1–MCV2 Dropout (%)**	**2023 Coverage Interpretation**
**Countries achieving ≥95% coverage for both doses**				
Bahrain	99	99	0	Sustained elimination-level coverage
Egypt	96	95	1	Sustained elimination-level coverage
Iran (Islamic Republic of)	99	99	0	Sustained elimination-level coverage
Morocco	98	98	0	Sustained elimination-level coverage
Oman	99	99	0	Sustained elimination-level coverage
Qatar	99	99	0	Sustained elimination-level coverage
Saudi Arabia	97	96	1	Sustained elimination-level coverage
Tunisia	96	97	−1.0	Sustained elimination-level coverage
**Countries with moderate coverage or MCV2 gaps**				
Jordan	95	89	6.3	Moderate MCV2 gap
Kuwait	99	91	8.1	Moderate MCV2 gap
United Arab Emirates	98	94	4.1	Moderate MCV2 gap
Iraq	97	83	14.4	Significant MCV2 gap
**Countries with suboptimal coverage (<95%)**				
Pakistan	84	80	4.8	Persistent immunity gaps
Occupied Palestinian territory	89	89	0	Coverage plateau
Libya	73	72	1.4	Suboptimal coverage
Syrian Arab Republic	74	64	13.5	Suboptimal coverage
Djibouti	76	64	15.8	Significant MCV2 gap
Lebanon	67	59	11.9	Persistent low coverage
**Countries with critical immunity gaps**				
Somalia	63	18	71.4	Severe dropout and low coverage
Afghanistan	55	42	23.6	Critical immunity gap
Sudan	51	38	25.5	Critical immunity gap
Yemen	45	39	13.3	Extremely low coverage
**Panel B. Immunization Coverage and Dropout, 2024**				
**Country**	**MCV1 (%)**	**MCV2 (%)**	**MCV1–MCV2 Dropout (%)**	**2024 Coverage Interpretation**
**Countries achieving ≥95% coverage for both doses**				
Bahrain	99	99	0	Sustained elimination-level coverage
Egypt	97	96	1	Sustained elimination-level coverage
Iran (Islamic Republic of)	99	99	0	Sustained elimination-level coverage
Morocco	98	98	0	Sustained elimination-level coverage
Oman	99	99	0	Sustained elimination-level coverage
Qatar	99	99	0	Sustained elimination-level coverage
Tunisia	96	97	−1.0	Sustained elimination-level coverage
**Countries with moderate coverage or MCV2 gaps**				
Jordan	99	96	3	Improved MCV2 coverage
Saudi Arabia	96	96	0	Near elimination threshold
Kuwait	98	94	4.1	Moderate MCV2 gap
United Arab Emirates	98	92	6.1	Moderate MCV2 gap
Iraq	91	82	9.9	Declining coverage
**Countries with suboptimal coverage (<95%)**				
Libya	89	80	10.1	Improved but suboptimal
Pakistan	86	82	4.7	Persistent immunity gaps
Syrian Arab Republic	81	75	7.4	Improving coverage
Occupied Palestinian territory	89	89	0	Coverage plateau
Lebanon	67	59	11.9	Persistent low coverage
Djibouti	75	48	36	Severe MCV2 dropout
**Countries with critical immunity gaps**				
Somalia	64	32	50	Severe dropout and low coverage
Afghanistan	55	44	20	Critical immunity gap
Sudan	46	36	21.7	Declining coverage
Yemen	41	40	2.4	Extremely low coverage

**Table 3:** Regional Performance Against WHO Measles and Rubella Elimination Indicators, WHO Eastern Mediterranean Region (EMR), 2023–2024.

Indicator	WHO Target	2023 *n* (%)	2024 *n* (%)
**Immunization Performance**			
Countries achieving ≥95% national coverage with two doses of measles-containing vaccine (MCV)	≥95%	8 (36%)	9 (41%)
**Surveillance Quality and Sensitivity**			
Countries achieving timely and complete investigation of suspected measles cases	≥80%	9 (41%)	11 (50%)
Countries with ≥80% of suspected cases having adequate specimens collected and tested in a proficient laboratory	≥80%	21 (95%)	20 (91%)
Countries reporting ≥80% of IgM laboratory results to national authorities within 4 days	≥80%	14 (64%)	11 (50%)
Countries achieving annualized discarded non-measles/non-rubella case rate ≥ 2 per 100,000 population	≥2	16 (73%)	15 (68%)
**Disease Burden and Elimination Status**			
Countries achieving endemic measles incidence < 1 case per 1,000,000 population	<1	0 (0%)	1 (6%)
Countries verified as having achieved or sustained measles/rubella elimination	—	3 (19%)	2 (13%)
Countries with ongoing endemic transmission or outbreaks	—	13 (81%)	14 (81%)

Abbreviations: EMR, Eastern Mediterranean Region; IgM, immunoglobulin M; MCV, measles-containing vaccine; WHO, World Health Organization. Note: Percentages were calculated using the total number of reporting countries and territories in the WHO EMR for each indicator year. Data sources: WHO/UNICEF Joint Reporting Form (JRF), case-based surveillance systems, and WHO regional databases.

## Data Availability

The data used in this study are derived from publicly available sources maintained by the World Health Organization. Measles and rubella surveillance data are accessible through the WHO Eastern Mediterranean Regional Office surveillance dashboard. National immunization coverage estimates are available through the WHO/UNICEF Estimates of National Immunization Coverage (WUENIC) at https://www.who.int/teams/immunization-vaccines-and-biologicals/immunization-analysis-and-insights/global-monitoring/immunization-coverage/who-unicef-estimates-of-national-immunization-coverage (accessed on 15 January 2026). Country-reported immunization data are available via the WHO/UNICEF Joint Reporting Form (eJRF) at https://immunizationdata.who.int (accessed on 15 January 2026). No new primary data were generated or analysed in this study.
